# Language Learners’ Attitudes Toward Online and Face-To-Face Language Environments

**DOI:** 10.3389/fpsyg.2022.926310

**Published:** 2022-07-19

**Authors:** Munassir Alhamami

**Affiliations:** English Department, King Khalid University, Abha, Saudi Arabia

**Keywords:** attitude toward behavior (ATB), social psychology, attitudes, learner academic engagement, beliefs, online and higher education

## Abstract

Learners’ attitudes are important for language learning. The attitude toward behavior construct, established in social psychology, was selected to elicit and examine learners’ attitudes toward face-to-face and online language learning. Data were collected using two instruments—questionnaires and interviews with two groups: face-to-face (*n* = 681) and online language learning (*n* = 287). The results show that the attitude toward behavior concept is an effective theoretical framework for designing questionnaires to understand the factors that influence the participants’ attitudes and to predict these in different learning environments. I compared the two groups’ results and found a more positive attitude toward language learning in face-to-face environments than in online language learning settings. The mixed-method design enables us to assess learners’ attitudes to the language learning environment. This informs curriculum design, policy, and support for teaching and learning.

## Introduction

Online language learning (OLL) is ubiquitous in the contemporary educational environment; hence, it is important to understand how learners’ attitudes toward OLL affect their behavior. Language learners’ attitudes toward the learning environment are a crucial factor affecting their behavior in classrooms (Ajzen and Fishbein, 2005). If a learner has a positive attitude toward a language-learning environment, they will put in more effort to learn and succeed. The inverse is also true: if learners pay more attention, they might have positive beliefs about the language-learning environment. Also, learners may find it difficult to interact with their classmates and teachers if they have a negative attitude toward the language-learning environment.

Language learners’ attitudes can be understood by directly asking them explicit questions and can be inferred from answers to questions about the important factors that influence their attitudes. Different factors can illustrate language learners’ attitudes, such as their intentions to interact and discuss with their classmates and instructors, ability to focus and pay attention during classes, and motivation to study and prepare for language lessons. Understanding these factors will help language instructors understand and predict their students’ general attitudes toward the learning process. Additionally, comparing learners’ attitudes toward the environment will reveal their preferences (for example, between online and face-to-face options). By comparing learners’ attitudes toward the environments, instructors will be able to explain a specific student’s behavior, such as low attendance and lack of engagement.

However, learners’ attitudes are complex and difficult to examine without the use of valid and reliable tools to elicit and analyze them. In this mixed-design study, I investigate the factors that influence language learners’ attitudes with regard to participating in sessions for learning English as a foreign language (EFL) in two settings: face to face language learning (FLL) and OLL. Ajzen’s Attitude toward Behavior (AB) concept (2002) is used to design the research tools for collecting and analyzing factors that influence the participants’ attitudes in FLL and OLL classes. The concept of attitude is firmly established in social psychology research (Eagly and Chaiken, 1993), on which Ajzen’s theory is based.

### **Theoretical** Framework

The concept of attitude has been investigated in various fields and is one of the central concepts of social psychology. Ajzen’s theory of planned behavior (PB) is one of the most influential theories to explain the relationship between attitude and human behavior, arguing that human social behavior is reasoned or planned. Individuals are assumed to consider a behavior’s expected consequences, the normative expectations of important people, and factors that may impede the performance of the behavior (Ajzen, 2007). The theory is designed to explain and predict human behavior and to provide a framework for devising future behavioral change interventions. According to the theory, human behavior is guided by three kinds of considerations: attitude toward behavior (AB), subjective norms, and perceived behavioral control (Ajzen, 2008).

I adopt Ajzen’s AB concept to investigate and compare language learners’ attitudes. AB is “the individual’s positive or negative evaluation of performing a particular behavior of interest” (Ajzen, 2005, p. 118). According to Ajzen (2006), an individual’s AB is the degree to which their performance of a certain behavior is positively or negatively valued through the lens of the expectancy-value model. AB is governed by a total set of accessible behavioral beliefs, linking the behavior to various outcomes and other attributes. Specifically, the strength of each belief (b) is weighted by the evaluation (e) of the outcome (i) or attribute, and the products are aggregated, as shown in the following equation:


(1) A⁢B∝Σ⁢b⁢i⁢e⁢i

**FIGURE 1:**
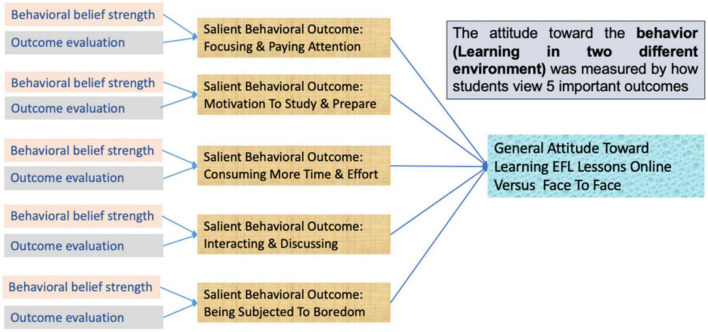
An illustrative example from this study.

According to Ajzen (2006), AB comprises the beliefs about the likely outcomes of the behavior and the evaluations of these outcomes (behavioral beliefs); in aggregate, these beliefs produce a positive or negative attitude toward the behavior. Ajzen (2015) explains that “the strength of each belief (b) is multiplied by the subjective evaluation (e) of the outcome (i), and the resulting products are summed. A person’s attitude toward the behavior is expected to be directly proportional (∝) to this summative belief composite” (p. 127). Figure 1 illustrates the equation to predict students’ attitudes toward learning English in two learning environments. Each salient behavior outcome, such as focusing and paying attention, should be measured by two different questionnaire items. One questionnaire item measures behavioral belief strength and another item measures the outcome evaluation of the same salient behavioral outcome. Measuring several outcomes will capture the general attitude. In each situation, there are important factors that can influence how and why certain attitudes form.

### Literature Review

#### Early Research on Language Learning and Attitudes

Early research clarifies the individual differences between successful and less successful learners (e.g., Fillmore et al., 1979) to understand learner attitudes. The early researchers do not focus on “attitude” but stress that it is important when identifying factors that help students become proficient in foreign languages.

Research on language-learner attitudes is also central to studies on motivation and anxiety in language learning classrooms. Gardner’s research on motivation and attitude is arguably a pioneering work that discusses language learners’ attitudes (1968). Gardner (1968) and his colleagues examined French learners’ attitudes toward learning French and English in Canada between the 1960s and 1970s. Attitudes and emotions are important aspects that enable us to understand factors such as students’ identity, behaviors, and performance.

#### Importance of Attitudes in Language Learning Studies

Language learning researchers (e.g., Thompson, 2021) stress that learners’ success or failure in language learning is affected by their attitudes. Language learners with positive attitudes persevere to achieve mastery of new languages, while negative attitudes hamper learning. Bai (2020) explores relationships between Chinese language learners’ attitudes toward the use of the College English Test—a high-stakes test in China—and their test performance. The results demonstrate that the students’ positive attitudes toward the use of the College English Test have a direct, significant, and positive impact on students’ test performances. In addition, Hu et al. (2021) add language learning boredom as a factor in the emerging discipline of language learning emotion research.

This is supported by the study by Zhu et al. (2020), which explores 94 college students’ online learning attitudes in a blended class. They examine the changes in the students’ attitudes toward online classes and the relationships between their self-regulated learning capability, online engagements, attitudes, and intentions. They find that the participants’ online learning attitudes and their attitudes toward face-to-face learning, substantially impact their intentions to undertake future online courses. Moreover, Szyszka (2020) finds a correlation between students’ positive attitudes and their pronunciation of the target language. Participants with positive attitudes are perceived as less accented.

#### Factors Correlated With Learners’ Attitudes

Factors that shape language learners’ attitudes reveal the complexity of investigating attitudes in online and face-to-face contexts. The literature illustrates that different researchers find different factors that influence learners’ attitudes. For example, previous educational experience in learning languages can determine a learner’s attitude toward learning languages (Dornyei, 2019). Additionally, Saito et al. (2018) assert that personal experience and experience with schooling and instruction are influential learning factors. They posit that attitudes and motivation toward learning are created through previous learning experiences.

Learners’ important references and role models are another important factor. Tao and Xu (2022) stress that parents’ opinions influence a student’s attitude toward language learning and can shape positive or negative attitudes in their children. Donitsa-Schmidt et al. (2004) report similar findings. They investigate whether changes in the educational context of teaching Arabic in Israeli schools affect the students’ attitudes toward the language and its speakers. Their results confirm the important role that parents have on their children’s behavior—they are a predictor of students’ motivation to study Arabic in the Israeli context. Teachers also play a significant role in forming their students’ beliefs about language learning. Ahmadi-Azad et al. (2020) and Kim (2021) argue that differences between the beliefs of students and teachers regarding language can negatively affect the students’ satisfaction with the language class, which might lead to the discontinuation of the student’s language learning journey.

#### Learners’ Attitudes Toward Technology

In computer-assisted language learning studies, researchers have found that language learners have positive attitudes toward the use of modern technologies—tablets (Chen, 2013), Facebook (Barrot, 2016), augmented reality (Wu, 2019), and digital games (Fu et al., 2019)—to learn languages. Learners’ positive attitudes toward digital technologies can facilitate their learning process in language classrooms (Arrosagaray et al., 2019; Faramarzi et al., 2019). To illustrate, Botero et al. (2018) find that students’ attitudes toward mobile language learning and acceptance of mobile devices are key factors in successful mobile learning implementation. In addition, positive attitudes are associated with the frequency of the use of technology to learn foreign languages. Su et al. (2019) stress that self-regulation and attitude are closely associated, because a high sense of self-regulation enhances learners’ attitudes or preferences toward online learning. Moreover, Hsu (2016) finds that learners’ attitudes are decisive in the sustained use of a computer-assisted pronunciation training system. Li et al. (2019) also report that language learners’ intentions to use automated writing evaluation software are directly determined by their perceptions of its usefulness, their attitudes toward using the system, and computer self-efficacy.

Various factors influence attitudes to the extent that it would be difficult for researchers to combine them in one study. For example, gender differences are a factor influencing attitudes toward technology use in education. Cai et al. (2017) conducted a meta-analysis of 50 articles from 1997 to 2014. The results show that male learners still hold more favorable attitudes toward technology use than female learners, but such differences are characterized as having a small effect size. However, Alothman et al. (2017) found no gender differences in the attitudes toward computer use among Saudi undergraduate students. Attitude is influenced by the city of study, parental encouragement, and English language proficiency, but not gender. Nevertheless, language learners’ attitudes can be changed and improved over time. Ianos et al. (2017) explore changes in attitudes toward languages over 2 years of secondary education of immigrants in Spain. Their findings indicate that it is possible to change attitudes and enhance positive attitudes. However, extensive effort is required to foster the development of positive attitudes.

Hou and Aryadoust (2021) identify language learning and modern technological instruments that reliably measure attitude. They find a lack of evidence for the accuracy of many of the measurement instruments used in language learning and argue that this prevents researchers from validating the instruments used to measure attitude. Therefore, valid and reliable measurements of learners’ attitudes can enable instructors to understand their learners and consequently provide the right treatment to improve the learners’ attitudes.

### Research Gap

Although many researchers (e.g., Cai et al., 2017; Thompson, 2021) found correlations between positive attitudes and success in achieving learning outcomes, the prediction of attitudes and factors that influence learners’ attitudes have not been fully explored in the literature, especially in educational technology studies. In this study, I used a valid and reliable instrument to predict learners’ attitudes and explore factors that influence language learners’ attitudes toward language learning environments. The results of this study contribute to filling the gap noted by several researchers (Gonulal, 2019; Hou and Aryadoust, 2021), who noticed that only a few computer-assisted language learning instruments that could reliably measure attitude had been developed and verified.

The literature review highlights the importance of understanding the attitudes of language learners since these influence their achievement of learning outcomes. Positive attitudes toward the learning environment motivate learners to spend more time studying and engaging in the learning process. Learners with negative attitudes toward the learning environment will not engage in the classroom or capitalize on the learning resources. By examining and understanding learners’ attitudes in FLL and OLL contexts, researchers will be able to suggest improvements to facilitate attitude changes.

### Research Questions

I build on previous research to investigate students’ AB, specifically students’ attitudes toward learning EFL in FLL and OLL contexts, referring to EFL students’ AB as the outcome against which attending and learning in FLL and OLL classes is assessed. The research questions are as follows:

(1) According to Ajzen’s AB construct, what are the factors that influence EFL learners’ attitudes?
(2) Based on Ajzen’s AB concept, do EFL learners have more positive attitudes toward FLL or OLL environments?
(3) To what extent does Ajzen’s AB concept help language educators and researchers understand language learners’ attitudes in FLL and OLL environments?

## Methodology

### Context

This study was conducted within an EFL program at a Saudi university. This program is offered to students in their first university year. The participants have different university majors, such as Engineering, Chemistry, and Biology. They all reside in Saudi Arabia and speak Arabic as their mother tongue. They are between 19 and 25 years of age. However, the instructors come from different cultures and countries (e.g., Algeria, Bangladesh, Britain, Canada, Egypt, India, Jordan, Pakistan, Romania, and Saudi Arabia). I considered ethical issues and obtained permission from the Institutional Review Board (IRB) before conducting the research. The participants submitted an informed consent form before they participated in the study. The informed consent form was translated into Arabic to ensure that they understood the details.

### Study Design

I employed a mixed-methods design to examine students’ attitudes through an iteration of connected quantitative and qualitative phases that are sequentially aligned. The design comprises a qualitative phase, followed by a quantitative phase, followed by a qualitative phase (i.e., qual → quan → qual). The rationale is based on the theoretical framework provided by Ajzen (2006) and the principle that it is difficult to investigate and understand attitudinal beliefs using one instrument in one phase. By using open-ended questionnaires, closed-ended questionnaires, and interviews in three phases, I was able to gain an in-depth understanding of the attitudinal beliefs of learners. The mixed-methods design enabled me to validate the results from one phase to another. Findings in each phase provide additional evidence and support for the findings in the next phase. Also, the qualitative data provided a detailed understanding of the context, and the quantitative data increased the reliability and generalizability of the results. The phases and participants included in each are presented in Table 1.

**TABLE 1:** Number of participants in the study.

Instruments	Eliciting study participants	Pilot study participants	Final study participants
Questionnaires	61 FLL group	99 FLL group	681 FLL group
	64 OLL group	70 OLL group	287 OLL group
Interviews	4 FLL group	N\A	22 FLL group
	4 OLL group	N\A	16 OLL group

Ajzen’s (2006) theory requires elicitation work to identify accessible behavioral beliefs. Therefore, in the first phase, I conducted two elicitation studies using interviews and open-ended surveys from two groups—OLL and FLL. In the latter group, 61 students responded to the open-ended survey, and four students completed the interview. In the online group, 64 students responded, and four students were interviewed. After analyzing the qualitative data from the questionnaires and the interviews of the FLL and OLL groups, the first drafts of the questionnaires for the online and face-to-face groups were designed and piloted. Next, a total of 99 students participated in the pilot study of the face-to-face questionnaire and 70 in the online questionnaire. Table 1 illustrates the number of participants in each phase of the study. In addition, nine instructors reviewed the two questionnaires. After revising the pilot questionnaires based on the comments and suggestions from the nine reviewers and participants in the pilot study, I designed the final questionnaires for the FLL and OLL groups and conducted the survey. Although no standard questionnaire uses the AB concept, some published samples such as those used by Ajzen (2006, 2013) and Fishbein and Ajzen (2010) provide a default reference for designing the final questionnaire. In addition, I utilized the guidelines and recommendations provided by Ajzen (2006) and Francis et al. (2004) for how to construct a theory of planned behavior questionnaire. Then, the FLL group experienced 2 weeks of reading lessons in face-to-face settings, and the OLL group experienced 2 weeks of reading lessons online. After that, interviews were conducted with the participants in the FLL and OLL groups. The interview participants were selected randomly from the FLL and OLL groups based on their willingness to participate in the interviews. Table 2 illustrates the timeline of the study.

**TABLE 2:** Steps and procedures of data collection, analysis, and interpretation.

1	Obtain the salient behavioral beliefs about OLL and FLL using open-ended surveys and interviews.
2	Analyze the data from the open-ended surveys and the interviews.
3	Design the pilot OLL and FLL groups’ questionnaires.
4	Pilot the OLL and FLL groups’ questionnaires.
5	Receive feedback about both pilot questionnaires from the reviewers and the results of the pilot studies.
6	Revise the pilot OLL and FLL groups’ questionnaires based on the feedback.
7	Design the final two questionnaires: OLL and FLL groups.
8	Conduct the final OLL and FLL groups’ questionnaires simultaneously.
9	Conduct 2 weeks of reading lessons for the OLL and FLL groups simultaneously.
10	Conduct interviews with participants from the OLL and FLL groups.
11	Analyze the quantitative data from the OLL and FLL groups’ questionnaires.
12	Analyze the qualitative data from the interviews and questionnaires’ open-ended questions for the FLL and OLL groups.
13	Interpret the quantitative and qualitative results for the FLL and OLL groups.
14	Presetting the results of FLL and OLL groups.

### Participants

The participants in the FLL group were students in the 011 Intensive English Program offered in the first semester of the first year (level one) for students who plan to join the Colleges of Engineering, Computer Science, Science, Financial and Administrative Sciences, Humanities, and Education. The program offered four courses: Listening, Grammar, Writing, and Reading. The reading course was selected for this study to avoid the influence of any language-skill differences on students’ beliefs and performance. Students might hold different beliefs regarding the structures and objectives of the course. In other words, students might have different attitudes toward technologies in a listening course than in a writing course. The instructors in the course taught two chapters from the textbook for 2 weeks in the FLL settings. The participants in the OLL group were students in the 012 Intensive English Program offered in the second semester of the first year (level two) for students who finished level one (i.e., 011 Intensive English Program). Like the FLL group, the reading course was selected to match the topic area used for the ENG 011 (FLL) group. Two chapters were selected to be taught online for 2 weeks, using the Learning Management System Blackboard (LMS/Bb).

## Materials and Instruments

### Questionnaire

The final questionnaire consisted of three sections for the FLL and OLL groups (biographical information, closed-ended questions, and open-ended questions). Each questionnaire had items that measure students’ attitudes explicitly (Direct measure of AB) and each questionnaire had five indirect measures that measure AB implicitly, i.e., explicit items ask directly about the participants’ AB and implicit items measure the participants’ AB indirectly. The five implicit measures were presented in two items. In other words, two questions were asked for each theme. For example, in AB, a behavioral belief (focusing and paying attention) was presented in one item, and its outcome evaluation was presented in another item. Table 3 presents the selected five themes (indirect measures of AB) from the first qualitative phase that were used in the questionnaire for the FLL group and OLL groups.

**TABLE 3:** Questionnaire themes that emerged from the first qualitative phase.

FLL group themes	OLL group themes
1. Focus on FLL reading lessons.	1. Focus on OLL reading lessons.
2. Motivation to study and prepare for FLL reading lessons	2. Motivation to study and prepare for OLL reading lessons.
3. Being subjected to boredom and feeling of responsibility in FLL reading lessons.	3. Being subjected to boredom and feeling of responsibility in OLL reading lessons.
4. The percentage of time and effort in FLL reading lessons.	4. The percentage of time and effort in OLL reading lessons.
5. Interacting and discussing with teachers and classmates in FLL reading lessons.	5. Interacting and discussing with students and teachers in OLL reading lessons.

### Analysis Procedures for the Quantitative Data

After screening the surveys and excluding 81 participants from the FLL group and 45 from the OLL group, for reasons such as incomplete surveys, lack of biographical information, and disengaged responses, I entered the data into an Excel spreadsheet. I excluded nine multivariate outliers that exceeded the Mahalanobis values from the FLL and OLL groups. This is effective for measuring the distance between a point and a distribution to detect anomalies.

I used Cronbach’s alpha to measure the internal consistency of four items for the AB direct measure construct. The Cronbach’s alpha results showed the acceptable reliability of the scale in AB constructs for both the FLL surveys (α = 0.83) and OLL surveys (α = 0.88). The Cronbach’s alpha measures reliability (internal consistency). Specifically, the data were assessed for the following assumptions: univariate outliers, multivariate outliers, normality, and multicollinearity. Once the assumptions were met, I conducted two regression tests.

### Analysis Procedures for the Qualitative Data

To understand the participants’ attitudes toward OLL and FLL classes, I conducted several interviews with students from the FLL and OLL groups (22 and 16, respectively). Additionally, I gathered comments, suggestions, and thoughts from the written surveys (134 and 58 students from the FLL and OLL groups, respectively). I transcribed the interviews and surveys, and coded the information for attitudes that motivate and demotivate students to attend classes and learn in the FLL and OLL groups. Coded data verified several attitudes that motivated or demotivated the students with regard to learning in both environments. Subsequently, these beliefs were grouped into six categories.

The guidelines for analyzing the qualitative data included Brown’s (2014) seven guidelines for analyzing qualitative data in mixed-methods studies. Before analyzing the data, I read the data several times to become familiar with it. Braun and Clarke (2013) stress that the analysis of qualitative data essentially begins with a process of “immersion” in the data. In this phase, the researcher familiarizes themselves with the dataset’s content to notice things that might be relevant to the research questions. I coded the data that are relevant to the research questions of this study, then recoded the data and looked for patterns between the codes. After that, I mapped out tentative patterns, organized and reorganized the categories that answer the research questions, and expanded the quantitative results. During the analysis process, I searched for connections and considered multiple perspectives.

## Results

### Results of the Face to Face Language Learning Group

The total number of participants in the FLL group is 681. The participants of the FLL group studied on two campuses. They were registered for 26 university majors. Most of the students majored in Mathematics (*n* = 60), Physics (*n* = 47), Chemistry (*n* = 46), Biology (*n* = 40), Accounting (*n* = 40), and Law (*n* = 36). The lowest major was Computer Engineering (*n* = 14). They attended different colleges, including College of Sciences (*n* = 193), College of Financial and Administrative Sciences (*n* = 160), College of Engineering (n = 145), College of Computer Sciences (*n* = 72), College of Humanities (*n* = 56), and College of Education (*n* = 55).

First, a correlation test between the scores of the five AB themes (AB1 = focusing on FLL English reading lessons; AB2 = motivation to study and prepare for FLL English reading lessons; AB3 = being subjected to boredom in FLL English reading lessons; AB4 = consuming more time and effort in FLL English reading lessons; AB5 = interacting and discussing with the teachers and classmates of English reading lessons in FLL learning settings) and the mean score of the AB direct measures using the Pearson correlation. The five indirect measures correlated with the direct measure mean. Low correlations between direct measures and implicit measures indicated that indirect measures were poorly constructed or did not adequately cover the breadth of the measured construct.

**FIGURE 2:**
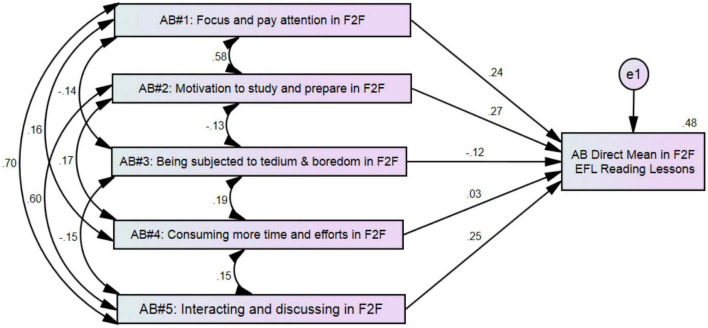
Attitude toward behavior (AB) diagram for the FLL group.

Each behavioral belief item was multiplied by its relevant outcome evaluation item in the survey. This created a new variable representing the weighted score for each behavioral belief. Next, the scores of the five AB implicit measures were used to predict the mean of AB explicit measures for the FLL group, using *n* = 681. Figure 2 is a diagram that illustrates the correlations among AB themes and shows the standardized effects of the five AB themes on the AB direct measures mean.

The five indirect variables, considered together, significantly predicted the AB direct mean (*p* < 0.00), with a 48% overlap between the five predictors and the outcome of AB. When predicting the AB direct measures mean, the test erred by approximately 0.67 AB-rating points based on a scale from 1 to 5.25. AB1, AB2, AB3, and AB5 remained significant predictors, with AB1 having a standardized direct effect of 0.24 (*p* = 0.00). AB2 had a standardized direct effect of 0.27 (*p* = 0.00), and AB3 had a standardized direct effect of –0.12 (*p* = 0.00). AB5 had a standardized direct effect of 0.25 (*p* = 0.00). Hence, AB1, AB2, AB3, and AB5 were significant predictors of the AB direct measure mean. AB4 was not a significant predictor of the AB direct mean, with a standardized direct effect of 0.03 (*p* = 0.36). A one-point increase in AB1, AB2, AB3, AB4, and AB5 was associated with an increase in the AB direct measure by 0.24 points, 0.27 points, 0.03 points, and 0.25 points, respectively. A one-point increase in AB3 was associated with a decrease in the AB direct measure by 0.12 points. Table 4 shows the descriptive statistics and correlation among variables.

**TABLE 4:** Descriptive statistics and correlations of attitude toward behavior (AB) for the FLL group (*n* = 681).

Variables		Pearson’s *r*					
	
	*M* (SD)	AB2	AB3	AB4	AB5	AB mean
AB1: focus and pay attention	3.91 (3.91)	0.58	–0.14	0.16	0.70	0.59
AB2: motivation to study and prepare	4.65 (4.26)		–0.13	0.17	0.60	0.58
AB3: being subjected to boredom	9.36 (7.30)			0.20	–0.15	–0.22
AB4: consuming more time and effort	6.12 (4.07)				0.15	0.13
AB5: interacting and discussing	4.22 (4.16)					0.60
Direct AB mean	2.16 (0.93)					

### Online Language Learning Group Results

The participants in the OLL group came from different academic backgrounds and were registered for 17 majors. Most of them majored in Accounting (*n* = 61), Business (*n* = 35), Electronic Marketing (*n* = 24). Few majored in Industrial Engineering (*n* = 8), and Mechanical Engineering (*n* = 8). The participants were from five colleges: College of Financial and Administrative Sciences (*n* = 164), College of Computer Sciences (*n* = 54), College of Engineering (*n* = 47), and College of Humanities (*n* = 22)

First, I used a correlation test to analyze the potential correlation between the scores of five AB themes (AB1 = Focusing and paying attention in online English reading lessons; AB2 = Having the motivation to study and prepare for online English reading lessons; AB3 = Having a feeling of responsibility for and high motivation toward online English reading lessons; AB4 = Saving time and efforts in online English reading lessons; AB5 = Interacting and discussing with students and teachers of English reading lessons in online settings), and the mean of the AB direct measures for the online group using Pearson’s *r*. The five themes correlated with the direct measure mean.

**FIGURE 3:**
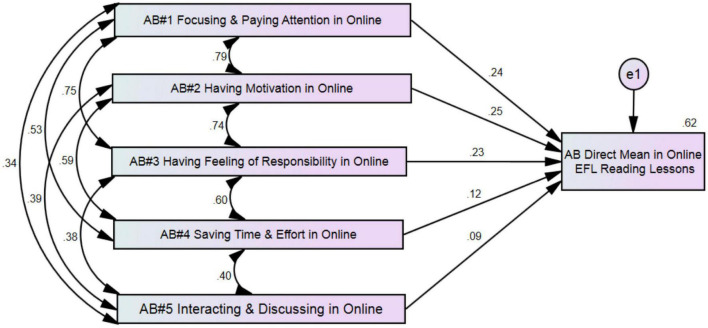
Attitude toward behavior (AB) diagram for the OLL group.

The scores of the five AB themes were used to predict the mean of AB direct measures (*n* = 287). Figure 3 is an AMOS diagram that illustrates the correlations among AB themes for the OLL group and shows the standardized effects of five AB themes on the AB direct measures mean for the OLL group.

The five variables, considered together, significantly predicted the AB direct measures mean (*p* < 0.00), with a 62% overlap between the five predictors and the outcome of AB. When predicting AB, the test erred by approximately 0.76 AB-rating points based on a scale from 1 to 6. AB1, AB2, AB3, AB4, and AB5 remained significant predictors, with AB1 having a standardized effect of 0.24 (*p* = 0.00), AB2 having a standardized effect of 0.25 (*p* = 0.00), AB3 having a standardized effect of 0.23 (*p* = 0.00), AB4 having a standardized effect of 0.12 (*p* = 0.02), and AB5 having a standardized effect of 0.09 (*p* = 0.03). Hence, AB1, AB2, AB3, AB4, and AB5 were all significant predictors of the AB direct measure mean.

A one-point increase in AB1, AB2, AB3, AB4, and AB5 was associated with an increase in the AB direct measure mean by 0.24 points, 0.25 points, 0.23 points, 0.12 points, and 0.09 points, respectively. Table 5 shows the descriptive statistics and correlation among variables.

**TABLE 5:** Descriptive statistics and correlations of attitude toward behavior (AB) for OLL group (*n* = 287).

Variables		Pearson’s *r*					
	
	*M* (SD)	AB2	AB3	AB4	AB5	AB mean
AB1 focusing and paying attention	6.69 (6.73)	0.79	0.75	0.53	0.34	0.70
AB2 being motivated to study/prepare	6.95 (6.89)		0.74	0.59	0.39	0.72
AB3 having a feeling of responsibility	6.69 (6.60)			0.60	0.38	0.70
AB4 saving time and effort	5.57 (5.77)				0.40	0.57
AB5 interacting and discussing	6.29 (5.01)					0.41
Direct AB mean	2.60 (1.22)					

### Comparing the Results of the Face to Face Language Learning and Online Language Learning Groups

The FLL group (*n* = 681) was associated with a direct AB score mean of *m* = 2.16 (*SD* = 0.93). By comparison, the OLL group (*n* = 287) was associated with a numerically higher AB mean m = 2.6 (*SD* = 1.2). To test the hypothesis that the FLL and OLL groups are associated with a statistically significantly different AB mean, I performed an independent sample *t*-test. The assumption of homogeneity of variances was not assumed via Levene’s F test, *F*_(431.7)_ = 34.41, *p* = 0.000. The independent sample t-test was associated with a statistically significant effect, *t*_(431.7)_ = -5.478, *p* = 0.001. Thus, the mean of the FLL group was determined to be statistically and significantly smaller than those in the OLL group. Cohen’s d was estimated at 0.41, which is a medium-size effect based on Cohen’s (1992) guidelines. Table 6 shows the descriptive statistics.

**TABLE 6:** Comparing the FLL and OLL groups.

	Group	*N*	Mean	SD	SE
AB mean	Face-to-Face	681	2.16	0.93	0.04
	Online	287	2.60	1.22	0.07

### Qualitative Results

#### Time

The first theme that emerged as a factor in participants’ attitudes is time. The participants express different attitudes toward the time of their classes. Most of them report that taking OLL classes saves their time and effort, while attending FLL classes consumes more time and effort. FLL students must commute to and from the campus. Some of the participants mentioned that they travel for about 1 h a day to attend their university classes. However, the flexibility in OLL classes might increase their procrastination to submit and attend OLL activities. The participants report that FLL classes have fixed times, thereby encouraging them to submit their class assignments on time and to be punctual. In addition, they state that it is faster to get an answer to their questions in FLL classes. They state that instructors often take a long time to respond to their questions in asynchronous OLL classes.

#### People

The second source of the participants’ positive and negative attitudes is the attitudes of people around them, such as teachers, classmates, relatives, friends, and future employers. Most of the people around the participants prefer taking FLL to OLL classes. Participants’ relatives and family members do not support taking OLL classes because they do not have any experience with this and think that FLL classes are more effective. Students also perceive that instructors have a more positive attitude in a face-to-face setting. The students stress that instructors who support FLL classes encourage students to take EFL classes in a face-to-face setting. In addition, the students report that classmates help one another with learning and translating during face-to-face classes. Specifically, their classmates can help them to understand instructions and perform class activities better because some are proficient in English. However, the participants report that OLL classes are more appropriate for their classmates who live far away from the campus. Taking OLL classes saves time and effort spent traveling or living in the city to attend classes.

#### Transportation

The third topic on which the participants have different attitudes is transportation when attending OLL and FLL classes. The length of transportation time and traffic problems prevent FLL students from regularly attending their classes at the university. Hence, the participants have a negative attitude toward going to campus during the rush hour, because it consumes more time and effort. In addition, they mention that they are frequently unable to find a taxi and that it is difficult to use public transport. They must use their cars or those of their friends or relatives for transport to the campus. The difficulty in attending FLL classes increases with distance. They also have problems finding convenient parking spots. These factors increase their negative attitudes toward attending FLL classes regularly and increase their preference for OLL classes.

#### Technology

Technology is the next factor around which the participants’ attitudes cluster. Participants stated that taking OLL classes can develop their computer skills and knowledge about using technology. OLL classes require students to know how to use a computer and some applications and learning management systems, such as Blackboard. Having pre-training in online learning before attending OLL classes decreases the participants’ negative attitudes toward OLL classes. However, the participants’ negative attitudes increase as the percentage of technical problems, such as Internet disconnection and maintenance of learning management systems, increases. OLL classes can help students who live in remote areas attend regularly and maintain a high percentage of attendance.

#### Educational Resources

The fifth construct concerns the participants’ attitudes toward the teaching methods and educational resources. The participants mention that teachers have more opportunities to use different and effective teaching methods in the FLL classes. The participants sometimes lack concentration and experience boredom during FLL classes. These increase their negative attitudes toward attending FLL classes. The participants state that their exam scores will be lower in OLL classes since they are not familiar with it. However, the participants stress that shy students will have a more positive attitude toward OLL classes because they can practice the language.

#### Communication

The next construct is communication—the participants believe that they have more opportunities to discuss and interact with their instructors and classmates in FLL compared with OLL classes. The participants report that they can use their gestures and body language to understand others and communicate with them. They also stress that the FLL environment provides more opportunities to ask questions, and encourage chats with classmates and instructors, compared to the OLL environment.

## Discussion

To answer RQ1—According to Ajzen’s AB construct, what are the factors that influence EFL learners’ attitudes?—I refer to Figures 2, 3, which present the important factors that influence learners’ attitudes. Figure 2 shows that all AB factors for the FLL group, except AB4 (consuming time and effort), significantly predict the AB direct measure mean. This means that AB1 (focusing and paying attention), AB2 (motivation to study and prepare), AB3 (fear of being subjected to boredom), and AB5 (interacting and discussing with teachers and classmates) are important factors in predicting students’ attitudes toward attendance and learning in the language lessons. However, AB4 (consuming time and effort in FLL EFL reading lessons) is not an influential factor in predicting students’ attitudes. The five variables were positively correlated with each other and with the attitude mean. However, AB3 negatively correlated with attitude mean and other attitude variables, because the fear of being subjected to boredom is a negative concept that contradicts other positive constructs such as paying attention, focus, motivation, interacting, and discussing. In other words, students have negative attitudes toward being subjected to boredom if they have positive attitudes toward the learning environment.

Hu et al. (2021) found that boredom in language classes is an important addition to the emerging field of foreign language learning emotion research. Although many factors influence language learners’ attitudes toward the learning environments, the results of this study found only five important factors (see Figures 2, 3 in two learning environments). Other researchers in different contexts found different important factors. For example, Donitsa-Schmidt et al. (2004) and Tao and Xu (2022) found a correlation between learners’ attitudes and that of their parents. Moreover, Ahmadi-Azad et al. (2020) and Kim (2021) found relationships between the beliefs of students and teachers. This difference might be due to the context of the study, learners’ ages, educational levels, and learners’ backgrounds.

Figure 3 shows that all AB themes of the OLL group significantly predict the AB direct measure mean for the OLL group. AB1 (focusing and paying attention), AB2 (being motivated to study and prepare), AB3 (having the feeling of responsibility), AB4 (saving time and effort), and AB5 (interacting and discussing with students and teachers) are considered important variables that influence students’ attitudes toward attendance of OLL classes. These results are limited to learners’ attitudes toward OLL using the Blackboard learning management system. Language learners’ attitudes might depend on the types of technology that instructors use in classrooms. For example, researchers in other contexts have found that students have positive attitudes toward other modern technologies such as Facebook (Barrot, 2016), augmented reality (Wu, 2019), and digital games (Fu et al., 2019). Language learners with positive attitudes toward technology will spend more time and focus more during online sessions. Hsu (2016) and Su et al. (2019) have stressed that participants’ attitudes are key factors for continuous language learning using modern technology.

To answer RQ2—based on Ajzen’s AB concept, do EFL learners have more positive attitudes toward FLL and OLL environments?—I compared the two groups. The comparison of the mean AB of the FLL and OLL groups reveals that the OLL group has negative attitudes toward OLL, based on the results of the five AB themes. Although many researchers encourage the use of online learning, the results of this study show that learners generally have negative attitudes toward OLL classes. This negative attitude will affect their language learning outcomes because having a positive attitude is crucial to succeeding in language learning environments (Botero et al., 2018). Language learners must have a strong positive attitude to independently overcome challenges in online learning. Participants’ familiarity with online learning and a higher level of computer knowledge seem to increase positive attitudes toward attending OLL classes. The participants’ previous experiences with OLL helped them to succeed in OLL classes. Negative attitudes toward the OLL environment might be a crucial factor that contributes to less success in achieving learning outcomes. Thompson (2021) emphasizes that learners’ success or failure in language learning is affected by their attitudes. Differences in the attitudes of the FLL and OLL groups can negatively affect the students’ satisfaction with the language class. Learners’ experiences and backgrounds play important roles in the results of this study. Saito et al. (2018) stress that learners’ personal experiences influence their attitudes toward the learning process. Learners’ attitudes are formed through many years of formal education. Differences in the attitudes of the FLL and OLL groups can negatively affect the students’ satisfaction with the language classes. Learners’ experiences and backgrounds play important roles in the results of this study.

To answer RQ3—To what extent might Ajzen’s AB concept help language educators and researchers understand language learners’ attitudes in FLL and OLL environments?—the findings of both quantitative and qualitative results demonstrate that Ajzen’s AB concept can help educators examine language learners’ attitudes. The high correlation among the themes in Figures 2, 3 shows the complexity of examining language learners’ attitudes. The use of Ajzen’s concept helps in understanding this phenomenon and analyzing the influence of attitudes in the FLL and OLL groups implicitly and explicitly. In addition, the use of a mixed-methods approach assists in understanding the learners’ attitudes toward both language learning environments. The use of a mixed-method design in this study helps us to understand the results in more detail and presents a clear image of language learners’ attitudes.

The results show the effective use of Ajzen’s AB concept and method of constructing the questionnaire items to predict participants’ attitudes. In turn, accurate prediction of attitudes will help teachers and researchers better understand language learners. Ajzen specifies the nature of relationships between beliefs and attitudes. The results show that learners’ attitudes toward learning the language in face-to-face and online settings are determined by their accessible beliefs about the learning environment. Specifically, the types of questionnaires on attitudes toward behavioral beliefs and outcome evaluations contribute to their attitudes in direct proportion. The strength of each belief concerning the outcome in the language learning environment and the evaluation of the outcome shape students’ language learning attitudes toward attending classes in this environment.

Overall, the results of qualitative data illustrate the quantitative results in more detail. The participants discussed the issues related to the five themes in Figures 2, 3. Moreover, qualitative data explains the results thoroughly. The six topics in the qualitative data around which the participants’ attitudes clustered help us understand the reasons that motivate students and increase their positive attitude toward the language learning environment. New changes in language learning environments, such as materials, teaching methods, and assessments, require understanding students’ attitudes toward these new changes. Successful changes in the language learning environment require successful changes in students’ attitudes to match the new orientations. The use of one approach will not help to understand attitudes in enough detail. Rather, the use of a mixed-method approach clarifies the attitudes and elucidates factors that form the participants’ attitudes toward the language learning environments.

Future studies might replicate this study in different contexts to validate the results and examine the use of the AB concept with different participants. Although the results of this study are limited to males, some studies (e.g., Alothman et al., 2017) found no difference between genders regarding attitudes toward using technology. The findings of Cai et al. (2017) show that males still hold more favorable attitudes toward technology use than female students. In this study, the participants (all males) hold more favorable attitudes toward FLL than OLL classes. Another limitation of the study is the subject of the course. Research might find different attitudinal beliefs based on the course content. Future studies might investigate learners’ attitudes using the same theoretical framework for different language learning skills, such as writing and listening. Students’ attitudes change over different periods of time; therefore, future studies might measure changes in learners’ attitudes since attitudes are not stable for long periods, especially regarding technology. Educational technologies are regularly updated; hence, learners’ attitudes might also change in response to this.

## Conclusion

There are several implications for the results of this study. First, the adaptation of a valid and reliable theoretical framework from another field—in this case, social psychology—proved effective for examining learners’ attitudes to different language learning environments. In this study, Ajzen’s AB concept is useful in understanding language learners’ attitudes toward a language-learning environment. Learners’ explicit attitudes toward their language-learning environment can be inferred from their attitudes toward other related factors, such as their attention, motivation, time, and interaction. Language educators must also use new, valid, and reliable theoretical concepts to analyze language learners’ attitudes and beliefs. Although language educators encourage the use of online learning, the results of this study show that to have a successful language learning environment, educators need to understand learners’ attitudes and examine the factors that influence such attitudes toward the language learning environment. Policymakers need to consider learners’ attitudes while designing curricula and learning materials.

## Data Availability Statement

Requests to access the datasets on which this article is based should be directed to MA, munassir7@gmail.com.

## Ethics Statement

The studies involving human participants were reviewed and approved by King Khalid University. The patients/participants provided their written informed consent to participate in this study.

## Author Contributions

The author confirms being the sole contributor of this work and has approved it for publication.

## Conflict of Interest

The author declares that the research was conducted in the absence of any commercial or financial relationships that could be construed as a potential conflict of interest.

## Publisher’s Note

All claims expressed in this article are solely those of the authors and do not necessarily represent those of their affiliated organizations, or those of the publisher, the editors and the reviewers. Any product that may be evaluated in this article, or claim that may be made by its manufacturer, is not guaranteed or endorsed by the publisher.
